# CBT for Childhood Anxiety: Reviewing the State of Personalised Intervention Research

**DOI:** 10.3389/fpsyg.2021.722546

**Published:** 2021-11-26

**Authors:** Lizél-Antoinette Bertie, Jennifer L. Hudson

**Affiliations:** ^1^Department of Psychology, Centre for Emotional Health, Macquarie University, Sydney, NSW, Australia; ^2^Black Dog Institute, University of New South Wales, Sydney, NSW, Australia

**Keywords:** cognitive-behavioral therapy, childhood anxiety, personalised interventions, predictor, moderator, mediator

## Abstract

This article presents a mini-review of the state of personalised intervention research in the field of child and adolescent anxiety. We evaluated narrative, systematic and meta-analytic reviews of key research methodologies and how they relate to current approaches for personalising CBT, specifically. Preliminary evidence of predictors (severity of primary disorder, social anxiety disorder (SoAD), comorbid depression, parental psychopathology, parental involvement and duration of treatment), moderators (type of primary disorder) and mediators (self-talk, coping, problem-solving and comorbid symptoms) of CBT outcomes provides content for several personalised approaches to treatment. Finally, we present a novel conceptual model depicting the state of personalised intervention research in childhood anxiety and propose a research agenda for continued progress.

## Introduction

For the past decade, personalised mental health intervention has been touted as the new frontier in clinical psychology. The notion that psychotherapy can be tailored to the needs of the individual is likewise gaining momentum in the field of childhood anxiety research (Ng and Weisz, 2016). As the most prevalent of childhood mental disorders affecting 15–20% of children, anxiety disorders lead to significant impairment across several domains of functioning and often follows a chronic course into adulthood (Polanczyk et al., 2015; Asselmann et al., 2018). At present, cognitive behavioural therapy (CBT) is the evidence-based treatment of choice producing positive results for approximately half of anxious children (James et al., 2020). The fact that nearly five out of 10 children still meet criteria for an anxiety disorder after treatment, along with the enormous individual, societal and economic burden of anxiety disorders (Kyu et al., 2016; Lee et al., 2017), underscores the need to understand and predict differential treatment response. It is crucial in personalising interventions in two ways: first, in matching the best treatment to an individual child and second, by developing new or modifying existing interventions (Simon and Perlis, 2010), which will both greatly benefit children and adolescents living with anxiety.

The movement towards personalised intervention is considered to be the answer to the question posed by Gordon Paul (1967): ‘*what* treatment, by *whom*, is most effective for *this* individual with *that* specific problem, and under *which* set of circumstances?’ Defined as evidence-based methods for tailoring treatments to individuals, personalised intervention implies that patient-specific features may guide a practitioner’s treatment decisions to optimise treatment outcome (Schneider et al., 2015; Ng and Weisz, 2016). Further, the three overarching goals of personalised intervention include making an accurate diagnosis, predicting individual risk and achieving an effective treatment response (Ozomaro et al., 2013). Despite substantial research efforts, evidence in support of predicting individual risk is inconsistent, and we still do not know how to improve outcomes for those children who do not optimally respond to treatment.

As the current ‘gold standard’ therapy, CBT is associated with considerable decreases in anxiety compared to control conditions at post-treatment, with good evidence of lasting changes at longer term follow-up (Gibby et al., 2017) and widespread positive outcomes across other functional areas (Kreuze et al., 2018). Further, CBT addresses anxiety through a core set of strategies comprising skill building based on psychoeducation about anxiety, somatic management strategies, cognitive restructuring techniques and gradual exposure to feared situations (Albano and Kendall, 2002). Consisting of strategies derived from cognitive and behavioural principles (Beck and Haigh, 2014), CBT has positioned itself as a prime candidate for personalisation. However, the questions of for whom, why and how this treatment works remain largely unanswered.

To better understand which children are most likely to benefit, and why, researchers have investigated predictors, moderators and mediators of treatment outcomes following CBT (Kraemer, 2013), with the focus on identifying the factors underlying successful response, or alternatively, the partial or lack of response from anxious children. Therefore, the objective of this mini-review was to evaluate existing research methodologies and current personalisation approaches that tailors CBT to treat child and adolescent anxiety.

## Predictors, Moderators and Mediators of Cbt Outcomes

A combination of narrative, systematic and meta-analytic reviews was identified and examined alongside relevant individual studies to evaluate the most prominent research methodologies currently employed in childhood anxiety research. To ensure we consulted the most recent evidence, we conducted a rapid review of the literature and identified 15 studies published in the last decade. Further information regarding the search strategy and inclusion criteria is presented in the online Supplementary Material. A summary of the studies and reported findings is discussed and presented in Table 1.

**Table 1:** Summary of predictors, moderators and mediators of CBT outcomes.

Design and study	Year	Type	Robust factors
**Predictor**			
Kunas et al.	2021	SR/MA	Primary AD severity
			Parental psychopathology
Perihan et al.	2020	SR/MA	Parental involvement
			Treatment duration
Gibby et al.	2017	SR	No robust predictors
Scaini et al.	2016	MA	Social skills training
Knight et al.	2014	SR	No robust predictors
Thulin et al.	2014	MA	No robust predictors
Nilsen et al.	2013	SR	No robust predictors
Mychailyszyn et al.	2012	MA	No robust predictors
**Moderator**			
Norris & Kendall	2021	NR	No robust moderators
Kreuze et al.	2018	MA	No robust moderators
Higa-McMillan et al.	2016	SR	Primary diagnosis
Ung et al.	2015	SR/MA	No robust moderators
Manassis et al.	2014	MA	No robust moderators
Bennett et al.	2013	IPDMA	No robust moderators
Nilsen et al.	2013	SR	No robust moderators
Mychailyszyn et al.	2012	MA	No robust moderators
**Mediator**			
Luo & McAloon	2021	MA	Externalising symptoms
			Depressive symptoms
			Self-talk (negative)
			Coping
Higa-McMillan et al.	2016	SR	Parental intrusiveness
			Post-event processing

*SR=systematic review, NR=narrative review, MA=meta-analysis, IPDMA = individual patient data meta-analysis*.

### Predictors

Most childhood anxiety research have investigated baseline characteristics that have a direct influence on how children respond to anxiety treatment, identifying predictors associated with treatment outcome independent of treatment modality (Kraemer et al., 2002). Reasons for the extensive predictor research evidence may include the availability of pre-treatment characteristics prior to treatment decisions being made, as well as the ease and low cost of data collection (Kunas et al., 2021). A number of systematic review and meta-review evaluated predictors of outcome following CBT across several RCTs which provided contradictory findings for several child demographic (age and gender), clinical (symptom severity and comorbidity) and parental factors (parental psychopathology; Mychailyszyn et al., 2012; Nilsen et al., 2013; Knight et al., 2014; Thulin et al., 2014). However, by utilising larger sample sizes, subsequent treatment studies identified a diagnosis of social anxiety disorder (SoAD), comorbid depression and parent psychopathology as more robust baseline predictors of poorer treatment response (Hudson et al., 2015). A recent systematic and meta-analytic review of predictors of youth anxiety and depression concluded that severity of the primary disorder and parental psychopathology significantly predicted negative CBT outcome for anxious children (Kunas et al., 2021). In contrast, some studies found that higher severity of the primary disorder predicted better response (i.e., decrease in anxiety symptoms; Kerns et al., 2013), while others reported poorer outcome (i.e., fewer children diagnosis free) at post-treatment and long-term follow-up (Gibby et al., 2017). Another systematic and meta-analytic review identified two treatment factors with results suggesting that increased parental involvement and longer duration of overall treatment were two robust factors associated with greater CBT effects (Perihan et al., 2020). Overall, the findings suggest that CBT is comparably effective for children and adolescents across all genders, ages, ethnicity and socio-economic status (Knight et al., 2014), and it may, however, point to the need to research latent factors that may have a direct influence on treatment outcome. Inconsistent predictor findings may also be ascribed to methodological issues, such as lack of statistical power, variations in methodology and variations in outcome measurement (response vs. remission), as possible reasons for not observing main effects across studies. Additionally, predictors fail to identify those who will benefit most from a given treatment and provide no recommendations for modification to treatment to optimise response (Kraemer, 2013) nor do they lend themselves to identifying processes that may serve as mechanisms for treatment outcome (Kraemer et al., 2002). Therefore, researching moderators and mediators of treatment outcome alongside predictors of outcome is paramount to improving the effectiveness of CBT by being able to personalise treatment (Huibers et al., 2021).

### Moderators

These factors refer to specific characteristics that predict greater benefit from one treatment over another to provide understanding for *whom* they may be effective (Kraemer et al., 2002). Despite considerable research effort, few variables have been identified as consistent moderators. Earlier systematic reviews of moderators of childhood anxiety and depression outcomes reported inconclusive moderation effects for the moderators under investigation (Mychailyszyn et al., 2012; Bennett et al., 2013; Nilsen et al., 2013; Manassis et al., 2014; Ung et al., 2015). Nilsen et al. (2013) noted that a lack of variability in the moderators may have complicated the comparison of results across studies as most studies primarily examined the efficacy of treatment. However, one systematic review reported a moderation effect for type of primary diagnosis (Higa-McMillan et al., 2016). Compton et al. (2014) examined potential moderating effects of primary anxiety diagnoses across four treatment conditions: anxiety medication sertraline (SRT), CBT, combined SRT+CBT and pill placebo. Results showed that youth with generalised anxiety disorder (GAD) demonstrated improved outcomes with CBT compared to SRT, whereas children with social anxiety disorder (SoAD) responded more favourably to treatment including SRT (combination and SRT alone) than CBT alone. A recent narrative review concluded that generally, no child demographic, clinical or parental characteristics consistently moderate treatment outcome (Norris and Kendall, 2021). Future research requires appropriate moderator study designs to identify the factors that robustly differentiate between treatments to assist in the clinician’s decision of which treatment is best for which child.

### Mediators

These factors identify critical processes and possible mechanisms through which treatment causes clinical change to understand *how* a treatment works (Kraemer et al., 2002). Regrettably, even fewer studies of potential mediators have been conducted for treatment outcome in childhood anxiety disorders, with little evidence in support of implying mechanistic change. CBT appears to be effective through content and process changes in relation to cognition and behaviour, as well as emotional and somatic outcomes (Herres et al., 2015). A recent systematic review and meta-analysis of mediators of CBT reported evidence for change in negative self-talk and coping, as well as change in depressive and externalising symptoms, as potential mechanisms (Luo and McAloon, 2021). Higa-McMillan et al. (2016) reported on mediators identified in studies and trials within their systematic review which showed that parental intrusiveness and post-exposure processing may be two further factors that mediate anxiety outcome. Further individual studies suggest that positive self-talk (Hogendoorn et al., 2014), coping self-efficacy (Kendall et al., 2016) and perceived control over anxiety (Marker et al., 2013) may also be potential cognitive mediators, while problem-solving and attention reallocation may represent behavioural mechanisms that increase coping (Hogendoorn et al., 2014). Questions remain regarding the effect of CBT on affective and physiological outcomes for children with anxiety, such as fear and physiological indicators of fear (Herres et al., 2015). The limited and unconvincing mediator findings have also been ascribed to the challenging nature of mediator research and insufficient methodologies, such as not demonstrating temporal precedence of the mediator (Huibers et al., 2021). Therefore, research with strong study designs to assess variables at multiple time points are required to delineate mechanisms of change (Luo and McAloon, 2021). Further, future research should consider the inclusion of a treatment comparison to examine effects of treatment components, for instance when findings show that participants experienced greater treatment effects when engaged in group CBT vs. individual CBT (Luo and McAloon, 2021). This is known as moderated mediation (Baron and Kenny, 1986), which provides us with information regarding potential mechanisms of change and for which children they may produce change.

## Personalised Intervention Approaches

**Figure 1:**
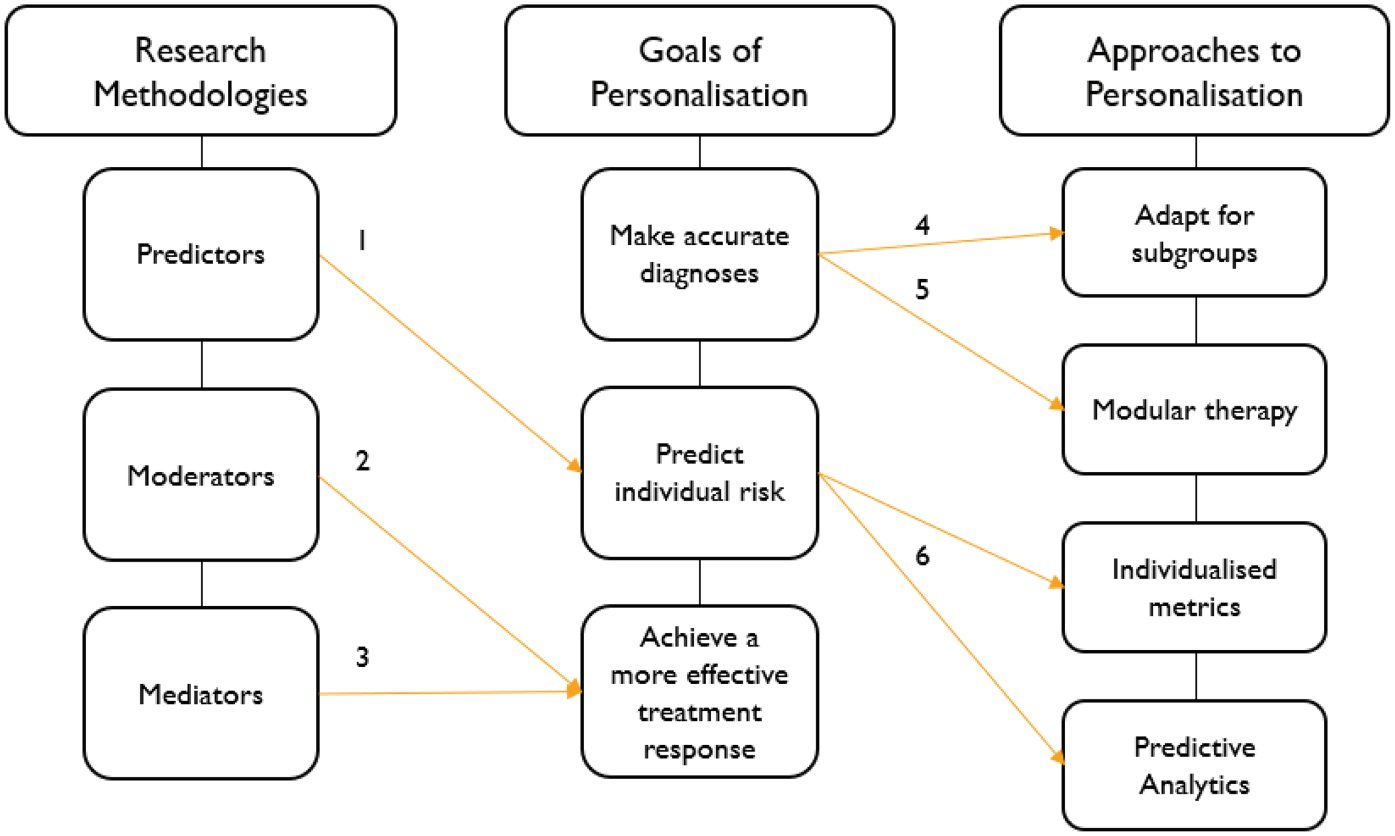
Conceptual Model of Personalised CBT for Childhood Anxiety. 1. Predictors predict risk of optimal/non-optimal response (i.e., parental psychopathology); 2. moderators predict benefit of one treatment over another for a subgroup of children (i.e., CBT over SRT for children with generalised anxiety disorder (GAD)); 3. mediators highlight mechanisms of change that influence outcome (i.e., reduce negative self-talk and increase coping abilities); 4 & 5. accurate diagnosis may facilitate subgroup and modular approaches (i.e., could children with SoAD benefit from additional social skills training or could anxious youth with comorbid depression benefit more from additional mood management modules?); and 6. understanding factors that predict individual risk facilitates the use of metrics and predictive analytics to inform treatment decisions (personalisation) to improve treatment outcomes.

In combination, predictor, moderator and mediator research align with the goals of personalising CBT intervention for childhood anxiety, for example, by identifying which factors predict risk of poorer treatment outcome, provide preliminary evidence of which CBT treatment factors may work best for a child with a certain risk profile and which mechanisms may be responsible for therapeutic change. Furthermore, these research methodologies also inform the development and testing of several personalised intervention approaches. A conceptual model depicting these associations is presented in Figure 1.

Ng and Weisz (2016) produced a comprehensive review of current strategies to personalised intervention for youth mental health, including approaches for which examples of CBT adaptation could be found. The most evaluated approach *adapts existing therapies for specific subgroups* that have been identified through predictor and moderator studies as at risk for poorer outcomes, for example children and adolescents with SoAD. Positive results have been demonstrated when using Social Effectiveness Therapy for Children (SET-C; Beidel et al., 2003), a group behaviour therapy program that specifically targets social deficits by combining social skills training, peer generalisation and individualised exposure. In an RCT examining the efficacy of SET-C compared to fluoxetine medication and pill placebo (Beidel et al., 2007), findings showed that both fluoxetine and SET-C outperformed placebo, but SET-C also enhanced social skills. This finding has been supported by a more recent meta-analysis reporting that when social skill training was included in treatment, it had an additional effect in reducing anxiety (Scaini et al., 2016).

A second approach is *modular therapy*. For instance, a child diagnosed with comorbid depression may receive modified treatment for anxiety by adding a module for mood management. An example is the Modular Approach to Therapy for Children with Anxiety, Depression, Trauma or Conduct Problems (MATCH) with treatment specifically targeting children who have one or a combination of these disorders (Chorpita and Weisz, 2009). An RCT conducted by Weisz et al. (2012) showed that modular therapy outperformed usual care and standard CBT with results indicating greater improvement and fewer diagnoses for children assigned to MATCH. Organising CBT into self-contained modules using individual or a combination of modules as required will contribute to a more flexible, dynamic and responsive treatment strategy (Ng and Weisz, 2016). More research is needed for empirically based methods to best select, combine and sequence modules for optimal treatment outcomes.

*Individualised metrics* offers a promising approach to personalised intervention, by quantifying the expected benefit each patient will receive, based on the child’s characteristics (Ng and Weisz, 2016). One example of an anxiety metric is the probability of treatment benefit (Lindhiem et al., 2012) modelled on the Child/Adolescent Anxiety Multimodal Study (CAMS) data set. This metric provided probabilities of experiencing improvement and positive outcomes for different levels of baseline severity and its interaction with treatment modality. It showed that children with severe baseline severity receiving a combination of SRT+CBT had a 62% probability of returning to normative anxiety, compared to 27% for SRT alone and 46% of CBT alone. However, children with moderate baseline severity had around 79% probability of returning to normative anxiety, regardless of treatment modality. While this study and its metric reported the effectiveness of CBT in terms of both response and outcome, it did not contain a control group to calculate differential probabilities, and further research on larger samples is required.

Another example of an individualised metric is a risk index, utilised as a clinical tool prior to treatment to identify children less likely to respond to standard CBT and who thus require modified intervention (Hudson et al., 2013). The researchers identified significant predictors of outcome and used their beta weights to calculate individual risk scores and examined the validity of the score to predict the likelihood of remission. The results showed that non-remission increased in a linear manner within each risk category, with 23% of low-risk scores (0–2) showing non-remission compared with 62% of high-risk scores (5–8). Future research is needed to replicate the results with larger samples and to include additional predictors of partial or non-remission.

Relatedly, another important approach represents the increasing interest in data and statistical driven methods to overcome several methodological difficulties on the road to personalised intervention. It is being argued that *predictive analytics*, such as machine learning methods, can integrate and make sense of bigger sets of healthcare data, because it is a natural extension to traditional statistical approaches (Beam and Kohane, 2018). Additionally, such methods have many advantages relative to linear models which is commonly used in mental health research (DeRubeis, 2019). For instance, machine learning methods can be used for multivariate model building with multidimensional psychological data and increases predictive ability while reducing overfitting of the model (Coutanche and Hallion, 2020). In sum, predictive analytics has the potential to facilitate personalised intervention in three ways: prediction of treatment response, supporting differential response and individual risk prediction (Hahn et al., 2017), providing increased incentive for its use in mental health.

## Barriers, Benefits and Future Directions: What Do We Need Now?

It is evident that clinical and research efforts to personalise interventions have the potential to significantly improve the lives of children with anxiety. Although the prospects are promising, this new frontier presents important challenges including generalisability of findings from the group to the individual-level (Norris and Kendall, 2021), implementation science (Williams and Beidas, 2019), extending access to care (Allen et al., 2020) and cultural adaptation of treatment (Naeem, 2019). However, it is the aforementioned methodological difficulties that remain the predominant challenge to the field. Valuable efforts have been made for standardising psychology research procedures to improve consistency and clarity in how RCTs and other treatment outcome studies are reported (Creswell et al., 2021). Further, the increase in childhood anxiety research over the past two decades created opportunities to combine data for a better understanding of differential treatment responses (e.g., The Genes for Treatment (GxT) study (Hudson et al., 2015)), along with the added benefits of increased statistical power and improved generalisability of findings (Lee, 2019). Further, methodological standardisation will facilitate meaningful synthesis of findings across studies when drawing conclusions regarding the extent to which CBT works for which children.

Considering the barriers, benefits and future directions of the childhood anxiety research, the field requires a strategic program of research that will bridge the gap between our current understanding of differential CBT response and the optimisation of treatment for young people at risk of poor outcome. Similar to a recently proposed agenda for personalising CBT for depression (Huibers et al., 2021), next steps should include the following: continued search for evidence of predictors, moderators and mediators and how they interact to affect change using large data sets and rigorous study methodologies, a considered research effort into the identification of treatment ingredients beyond common factors and their impact on therapeutic change (Norris and Kendall, 2021) and continued development and testing of modified CBT interventions in RCTs with strong control conditions.

This mini-review provides an evaluation of recent literature on current research methodologies, as well as approaches to the personalisation of CBT for childhood anxiety. A rapid review of the most recent narrative, systematic and meta-analytic reviews provided empirical support for the novel conceptual model that presents the associations between existing research methodologies, the goals of personalisation and current person-centred CBT treatment for childhood anxiety. Limitations include the evaluation of only a few approaches to personalising CBT, that is, there may be more potentially viable approaches and examples that were not considered given the limited scope of a mini-review.

## Conclusion

The process of personalised intervention for childhood anxiety is complex and enormous in scope. Clinical psychology research has made substantial progress in addressing differential CBT response within the context of childhood anxiety, producing evidence-based research strategies and approaches to personalising interventions. While the field has much to do to address persistent methodological challenges, rich opportunities exist for tailoring both treatment content and delivery to increase access to evidence-based care. With increasing collaboration among clinical researchers resulting in larger sample sizes, future research should consider the exciting yet untapped potential of predictive analytics to enhance personalisation efforts. This mini-review provides a novel explication of current research methodologies that provide content for personalised interventions with clinical relevance. Further, this review provides the first known conceptual model of personalised intervention research in childhood anxiety, while also supporting a call for a research agenda that is aligned with the goals of personalisation. Overall, the grand challenge for researchers remains to find innovative methods to personalise CBT interventions, which holds potential to significantly reduce the burden for children and adolescents living with anxiety disorders.

## Author Contributions

L-AB contributed to conception and wrote the first draft of the manuscript. JH contributed to manuscript revision and editing. All authors contributed to the article and approved the submitted version.

## Conflict of Interest

The authors declare that the research was conducted in the absence of any commercial or financial relationships that could be construed as a potential conflict of interest.

## Publisher’s Note

All claims expressed in this article are solely those of the authors and do not necessarily represent those of their affiliated organizations, or those of the publisher, the editors and the reviewers. Any product that may be evaluated in this article, or claim that may be made by its manufacturer, is not guaranteed or endorsed by the publisher.

## References

[ref1] Albano A. M. Kendall P. C. (2002). Cognitive behavioural therapy for children and adolescents with anxiety disorders: clinical research advances. Int. Rev. Psychiatry 14, 129–134. doi: 10.1080/09540260220132644

[ref2] Allen K. B. Benningfield M. Blackford J. U. (2020). Childhood anxiety—If we know so much, why are we doing so little? JAMA Psychiat. 77, 887–888. doi: 10.1001/jamapsychiatry.2020.0585, PMID: 32401291

[ref3] Asselmann E. Wittchen H. U. Lieb R. Beesdo-Baum K. (2018). Sociodemographic, clinical, and functional long-term outcomes in adolescents and young adults with mental disorders. Acta Psychiatr. Scand. 137, 6–17. doi: 10.1111/acps.12792, PMID: 28861892

[ref4] Baron R. M. Kenny D. A. (1986). The moderator-mediator variable distinction in social psychological research: conceptual, strategic, and statistical considerations. J. Pers. Soc. Psychol. 51, 1173–1182. doi: 10.1037//0022-3514.51.6.1173, PMID: 3806354

[ref5] Beam A. L. Kohane I. S. (2018). Big data and machine learning in health care. JAMA 319, 1317–1318. doi: 10.1001/jama.2017.18391, PMID: 29532063

[ref6] Beck A. T. Haigh E. A. (2014). Advances in cognitive theory and therapy: the generic cognitive model. Annu. Rev. Clin. Psychol. 10, 1–24. doi: 10.1146/annurev-clinpsy-032813-153734, PMID: 24387236

[ref7] Beidel D. C. Turner S. M. Morris T. L. (2003). Social Effectiveness Therapy for Children and Adolescents (SET-C): Therapist Guide. MHS.

[ref8] Beidel D. C. Turner S. M. Sallee F. R. Ammerman R. T. Crosby L. A. Pathak S. (2007). SET-C versus fluoxetine in the treatment of childhood social phobia. J. Am. Acad. Child Adolesc. Psychiatry 46, 1622–1632. doi: 10.1097/chi.0b013e318154bb57, PMID: 18030084

[ref9] Bennett K. Manassis K. Walter S. D. Cheung A. Wilansky-Traynor P. Diaz-Granados N.. (2013). Cognitive behavioral therapy age effects in child and adolescent anxiety: an individual patient data meta-analysis. Depress. Anxiety, 30, 829–841. doi:10.1002/da.22099, PMID: 23658135 PMC4854623

[ref10] Chorpita B. F. Weisz J. R. (2009). MATCH-ADTC: modular approach to therapy for children with anxiety, depression, trauma, or conduct problems. PracticeWise, LLC.

[ref11] Compton S. N. Peris T. S. Almirall D. Birmaher B. Sherrill J. Kendall P. C.. (2014). Predictors and moderators of treatment response in childhood anxiety disorders: results from the CAMS trial. J. Consult. Clin. Psychol. 82, 212–224. doi: 10.1037/a0035458, PMID: 24417601 PMC4056442

[ref12] Coutanche M. N. Hallion L. S. (2020). “Machine learning for clinical psychology and clinical neuroscience,” in Research Methods in Clinical Psychology. eds. Wright A. Hallquist M. (United Kingdom: Cambridge University Press), 467–482.

[ref13] Creswell C. Nauta M. H. Hudson J. L. March S. Reardon T. Arendt K.. (2021). Research review: recommendations for reporting on treatment trials for child and adolescent anxiety disorders—An international consensus statement. J. Child Psychol. Psychiatry 62, 255–269. doi: 10.1111/jcpp.13283, PMID: 32683742

[ref500] DeRubeis R. J. (2019). The history, current status, and possible future of precision mental health. Behav. Res. Ther. 123:103506. doi: 10.1016/j.brat.2019.103506 31706160

[ref14] Gibby B. A. Casline E. P. Ginsburg G. S. (2017). Long-term outcomes of youth treated for an anxiety disorder: A critical review. Clin. Child Fam. Psychol. Rev. 20, 201–225. doi: 10.1007/s10567-017-0222-9, PMID: 28181040

[ref15] Hahn T. Nierenberg A. A. Whitfield-Gabrieli S. (2017). Predictive analytics in mental health: applications, guidelines, challenges and perspectives. Mol. Psychiatry 22, 37–43. doi: 10.1038/mp.2016.201, PMID: 27843153

[ref16] Herres J. Cummings C. Swan A. Makover H. Kendall P. C. (2015). “Moderators and mediators of treatments for youth with anxiety,” in Moderators and Mediators of Youth Treatment Outcomes. eds. Maric M. Prins P. Ollendick T. (New York: Oxford University Press), 20.

[ref17] Higa-McMillan C. K. Francis S. E. Rith-Najarian L. Chorpita B. F. (2016). Evidence base update: 50 years of research on treatment for child and adolescent anxiety. J. Clin. Child Adolesc. Psychol. 45, 91–113. doi: 10.1080/15374416.2015.1046177, PMID: 26087438

[ref18] Hogendoorn S. M. Prins P. J. Boer F. Vervoort L. Wolters L. H. Moorlag H.. (2014). Mediators of cognitive behavioral therapy for anxiety-disordered children and adolescents: cognition, perceived control, and coping. J. Clin. Child Adolesc. Psychol. 43, 486–500. doi: 10.1080/15374416.2013.807736, PMID: 23795885

[ref19] Hudson J. L. Keers R. Roberts S. Coleman J. R. I. Breen G. Arendt K.. (2015). Clinical predictors of response to cognitive-behavioral therapy in pediatric anxiety disorders: The genes for treatment (GxT) study. J. Am. Acad. Child Adolesc. Psychiatry, 54, 454–463. doi:10.1016/j.jaac.2015.03.018, PMID: 26004660 PMC4469376

[ref20] Hudson J. L. Lester K. J. Lewis C. M. Tropeano M. Creswell C. Collier D. A.. (2013). Predicting outcomes following cognitive behaviour therapy in child anxiety disorders: the influence of genetic, demographic and clinical information. J. Child Psychol. Psychiatry 54, 1086–1094. doi: 10.1111/jcpp.12092, PMID: 23772677

[ref21] Huibers M. J. H. Lorenzo-Luaces L. Cuijpers P. Kazantzis N. (2021). On the road to personalized psychotherapy: A research agenda based on cognitive behavior therapy for depression. Front. Psych. 11:607508. doi: 10.3389/fpsyt.2020.607508, PMID: 33488428 PMC7819891

[ref22] James A. C. Reardon T. Soler A. James G. Creswell C. (2020). Cognitive behavioural therapy for anxiety disorders in children and adolescents. Cochrane Database Syst. Rev. 2020. doi: 10.1002/14651858.CD013162.pub2, PMID: 33196111 PMC8092480

[ref23] Kendall P. C. Cummings C. M. Villabo M. A. Narayanan M. K. Treadwell K. Birmaher B.. (2016). Mediators of change in the child/adolescent anxiety multimodal treatment study. J. Consult. Clin. Psychol. 84, 1–14. doi: 10.1037/a0039773, PMID: 26460572 PMC4695375

[ref24] Kerns C. M. Read K. L. Klugman J. Kendall P. C. (2013). Cognitive behavioral therapy for youth with social anxiety: differential short and long-term treatment outcomes. J. Anxiety Disord. 27, 210–215. doi: 10.1016/j.janxdis.2013.01.009, PMID: 23474911

[ref25] Knight A. McLellan L. Hudson J. L. Jones M. (2014). Pre-treatment predictors of outcome in childhood anxiety disorders: a systematic review. Psychopathol. Rev. a1, 77–129. doi: 10.5127/pr.034613

[ref26] Kraemer H. C. (2013). Discovering, comparing, and combining moderators of treatment on outcome after randomized clinical trials: a parametric approach. Stat. Med. 32, 1964–1973. doi: 10.1002/sim.5734, PMID: 23303653

[ref27] Kraemer H. C. Wilson G. T. Fairburn C. G. Agras W. S. (2002). Mediators and moderators of treatment effects in randomized clinical trials. Arch. Gen. Psychiatry 59, 877–883. doi: 10.1001/archpsyc.59.10.877, PMID: 12365874

[ref28] Kreuze L. J. Pijnenborg G. H. M. de Jonge Y. B. Nauta M. H. (2018). Cognitive-behavior therapy for children and adolescents with anxiety disorders: A meta-analysis of secondary outcomes. J. Anxiety Disord. 60, 43–57. doi: 10.1016/j.janxdis.2018.10.005, PMID: 30447493

[ref29] Kunas S. L. Lautenbacher L. M. Lueken U. Hilbert K. (2021). Psychological predictors of cognitive-behavioral therapy outcomes for anxiety and depressive disorders in children and adolescents: A systematic review and meta-analysis. J. Affect. Disord. 278, 614–626. doi: 10.1016/j.jad.2020.09.092, PMID: 33035949

[ref30] Kyu H. H. Pinho C. Wagner J. A. Brown J. C. Bertozzi-Villa A. Charlson F. J.. (2016). Global and National Burden of diseases and injuries Among children and adolescents Between 1990 and 2013: findings from the global burden of disease 2013 study. JAMA Pediatr. 170, 267–287. doi: 10.1001/jamapediatrics.2015.4276, PMID: 26810619 PMC5076765

[ref31] Lee Y. C. Chatterton M. L. Magnus A. Mohebbi M. Le L. K. Mihalopoulos C. (2017). Cost of high prevalence mental disorders: findings from the 2007 Australian National Survey of mental health and wellbeing. Aust. N. Z. J. Psychiatry 51, 1198–1211. doi: 10.1177/0004867417710730, PMID: 28565923

[ref32] Lee Y. H. (2019). Strengths and limitations of meta-analysis. Korean J. Intern. Med. 94, 391–395. doi: 10.3904/kjm.2019.94.5.391

[ref33] Lindhiem O. Kolko D. J. Cheng Y. (2012). Predicting psychotherapy benefit: A probabilistic and individualized approach. Behav. Ther. 43, 381–392. doi: 10.1016/j.beth.2011.08.004, PMID: 22440073 PMC3487390

[ref35] Luo A. McAloon J. (2021). Potential mechanisms of change in cognitive behavioral therapy for childhood anxiety: a meta-analysis. Depress Anxiety 38, 220–232. doi: 10.1002/da.23116, PMID: 33225527

[ref36] Manassis K. Lee T. C. Bennett K. Zhao X. Y. Mendlowitz S. Duda S.. (2014). Types of parental involvement in CBT with anxious youth: A preliminary meta-analysis. J. Consult. Clin. Psychol. 82, 1163–1172. doi: 10.1037/a0036969, PMID: 24841867

[ref37] Marker C. D. Comer J. S. Abramova V. Kendall P. C. (2013). The reciprocal relationship between alliance and symptom improvement across the treatment of childhood anxiety. J. Clin. Child Adolesc. Psychol. 42, 22–33. doi: 10.1080/15374416.2012.723261, PMID: 23009693 PMC4224949

[ref38] Mychailyszyn M. P. Brodman D. M. Read K. L. Kendall P. C. (2012). Cognitive-behavioral school-based interventions for anxious and depressed youth: a meta-analysis of outcomes. Clin. Psychol. Sci. Pract. 19, 129–153. doi: 10.1111/j.1468-2850.2012.01279.x

[ref39] Naeem F. (2019). Cultural adaptations of CBT: a summary and discussion of the special issue on cultural adaptation of CBT. Cogn. Behav. Ther. 12:e40. doi: 10.1017/S1754470X19000278

[ref41] Ng M. Y. Weisz J. R. (2016). Annual research review: building a science of personalized intervention for youth mental health. J. Child Psychol. Psychiatry 57, 216–236. doi: 10.1111/jcpp.12470, PMID: 26467325 PMC4760855

[ref42] Nilsen T. S. Eisemann M. Kvernmo S. (2013). Predictors and moderators of outcome in child and adolescent anxiety and depression: a systematic review of psychological treatment studies. Eur. Child Adolesc. Psychiatry 22, 69–87. doi: 10.1007/s00787-012-0316-3, PMID: 22923065

[ref43] Norris L. A. Kendall P. C. (2021). Moderators of outcome for youth anxiety treatments: current findings and future directions. J. Clin. Child Adolesc. Psychol. 50, 450–463. doi: 10.1080/15374416.2020.1833337, PMID: 33140992 PMC8089117

[ref44] Ozomaro U. Wahlestedt C. Nemeroff C. B. (2013). Personalized medicine in psychiatry: problems and promises. BMC Med. 11, 132. doi: 10.1186/1741-7015-11-132, PMID: 23680237 PMC3668172

[ref45] Paul G. L. (1967). Strategy of outcome research in psychotherapy. J. Consult. Psychol. 31, 109–118. doi: 10.1037/h0024436, PMID: 5342732

[ref46] Perihan C. Burke M. Bowman-Perrott L. Bicer A. Gallup J. Thompson J.. (2020). Effects of cognitive behavioral therapy for reducing anxiety in children with high functioning ASD: A systematic review and meta-analysis. J. Autism Dev. Disord. 50, 1958–1972. doi: 10.1007/s10803-019-03949-7, PMID: 30810842

[ref47] Polanczyk G. V. Salum G. A. Sugaya L. S. Caye A. Rohde L. A. (2015). Annual research review: A meta-analysis of the worldwide prevalence of mental disorders in children and adolescents. J. Child Psychol. Psychiatry 56, 345–365. doi: 10.1111/jcpp.12381, PMID: 25649325

[ref48] Scaini S. Belotti R. Ogliari A. Battaglia M. (2016). A comprehensive meta-analysis of cognitive-behavioral interventions for social anxiety disorder in children and adolescents. J. Anxiety Disord. 42, 105–112. doi: 10.1016/j.janxdis.2016.05.008, PMID: 27399932

[ref49] Schneider R. L. Arch J. J. Wolitzky-Taylor K. B. (2015). The state of personalized treatment for anxiety disorders: A systematic review of treatment moderators. Clin. Psychol. Rev. 38, 39–54. doi: 10.1016/j.cpr.2015.02.004, PMID: 25795293

[ref50] Simon G. E. Perlis R. H. (2010). Personalized medicine for depression: can we match patients with treatments? Am. J. Psychiatr. 167, 1445–1455. doi: 10.1176/appi.ajp.2010.09111680, PMID: 20843873 PMC3723328

[ref51] Thulin U. Svirsky L. Serlachius E. Andersson G. Ost L. G. (2014). The effect of parent involvement in the treatment of anxiety disorders in children: a meta-analysis. Cogn. Behav. Ther. 43, 185–200. doi: 10.1080/16506073.2014.923928, PMID: 24950054

[ref52] Ung D. Selles R. Small B. J. Storch E. A. (2015). A systematic review and meta-analysis of cognitive-behavioral therapy for anxiety in youth with high-functioning autism Spectrum disorders. Child Psychiatry Hum. Dev. 46, 533–547. doi: 10.1007/s10578-014-0494-y, PMID: 25246292

[ref53] Weisz J. R. Chorpita B. F. Palinkas L. A. Schoenwald S. K. Miranda J. Bearman S. K.. (2012). Testing standard and modular designs for psychotherapy treating depression, anxiety, and conduct problems in youth: A randomized effectiveness trial. Arch. Gen. Psychiatry 69, 274–282. doi: 10.1001/archgenpsychiatry.2011.147, PMID: 22065252

[ref54] Williams N. J. Beidas R. S. (2019). Annual research review: The state of implementation science in child psychology and psychiatry: a review and suggestions to advance the field. J. Child Psychol. Psychiatry 60, 430–450. doi: 10.1111/jcpp.12960, PMID: 30144077 PMC6389440

