# Supplementary material for: CBT for Childhood Anxiety: Reviewing the State of Personalised Intervention Research
Source: Front Psychol. 2021 Nov 26;12:722546. doi: 10.3389/fpsyg.2021.722546 (PMC8663921; doi:10.3389/fpsyg.2021.722546)
Supplement: Supplementary file 1 [file Data_Sheet_1.PDF]

Search Strategy for Mini Review

Inclusion / Exclusion criteria

Population:	Children and adolescents with a primary anxiety disorder diagnosis based on DSM criteria.
Age:	5 - 18 years
Anxiety disorder:	Generalised Anxiety (GAD), Separation Anxiety (SAD), Social Anxiety (SoAD), Specific Phobia (SP)
Intervention:	Cognitive behavioural therapy (CBT) and related components (i.e., exposure)
	Include: Treatment or intervention
	Exclude: Prevention studies
Outcomes:	Remission (clinical diagnosis of primary or all anxiety disorders),
	Response (change in pre-treatment primary severity)
	Functional outcomes
Article type	Only published studies as per Frontiers guidelines
	Include: Narrative reviews, systematic reviews, meta-analytic reviews
	Exclude: Grey literature
Language	Limit to English language only

Other considerations

Dates:	As the mini review focusses on recent developments in the field, we limited eligible reviews to the past decade.
	Exclude: reviews conducted before 2011
Database:	APA PsychInfo (ProQuest)
Search completed:	12 October 2021
Additional sources:	Screening reference lists

Search terms

cognitive behavio* OR cognitive therap* OR behavio* therap* OR exposure therap* OR cbt OR treatment OR intervention
AND anxiety disorder* OR anxi* OR general* anxiety OR social anxiety OR separation anxiety OR social phobi* OR specific phobi*
AND child* OR teen* OR adolesc* OR youth OR young pe* OR juvenil* OR school-age*
AND narrative review OR systematic review OR meta-analysis OR mini review

Search results

Raw results		215
Less: Limits applied	Scholarly Articles	-25
	English language	-5
	Age	-136
	**PsychInfo**	**49**
Additional records identified		22
	**Records screened**	**71**
Records excluded	Incorrect outcome: Not primarily anxiety	-19
	Incorrect treatment: Not CBT specific	-12
	Prevention study	-2
	Date published	-9
	Not predictor, moderator, or mediator studies	-12
	Incorrect age	-2
	**Records excluded**	**-56**
	**Reviews consulted**	**15**
